# Diet Quality and Measures of Sarcopenia in Developing Economies: A Systematic Review

**DOI:** 10.3390/nu14040868

**Published:** 2022-02-18

**Authors:** Amutha Ramadas, Hian Hui Law, Raanita Krishnamoorthy, Jordan Wei Shan Ku, Parimala Mohanty, Matteus Zhen Chien Lim, Sangeetha Shyam

**Affiliations:** 1 Jeffrey Cheah School of Medicine and Health Sciences, Monash University Malaysia, Jalan Lagoon Selatan, Bandar Sunway 47500, Malaysia; amutha.ramadas@monash.edu (A.R.); hlaw0007@student.monash.edu (H.H.L.); jkuu0001@student.monash.edu (J.W.S.K.); mlim0011@student.monash.edu (M.Z.C.L.); 2 Department of Social and Preventive Medicine, Faculty of Medicine, Universiti Malaya, Jalan Profesor Diraja Ungku Aziz, Kuala Lumpur 50603, Malaysia; raanita.k@gmail.com; 3 Department of Community Medicine, Institute of Medical Sciences & SUM Hospital, Siksha ‘O’ Anusandhan (Deemed to be University), K8 Lane, Kalinganagar, Bhubaneswar 751003, India; drparimalamohanty@gmail.com; 4 Division of Nutrition and Dietetics, School of Health Sciences, International Medical University, Jalan Jalil Perkasa 19, Bukit Jalil, Kuala Lumpur 57000, Malaysia; 5 Centre for Translational Research, IMU Institute for Research and Development (IRDI), International Medical University, Jalan Jalil Perkasa 19, Bukit Jalil, Kuala Lumpur 57000, Malaysia

**Keywords:** diet quality, sarcopenia, muscle loss, muscle strength, physical performance

## Abstract

Sarcopenia refers to common age-related changes characterised by loss of muscle mass, strength, and physical performance that results in physical disability, poorer health status, and higher mortality in older adults. Diet quality is indicated as a potentially modifiable risk factor for sarcopenia. However, the association between diet quality and sarcopenia in developing economies appears to be conflicting. Hence, we conducted a systematic review of the literature from developing economies examining the relationship between diet quality and at least one of the three components of sarcopenia, including muscle mass, muscle strength, and physical performance, and the overall risk of sarcopenia. No restrictions on age and study design were employed. We identified 15 studies that met review inclusion criteria. There was heterogeneity among the studies in the diet quality metric used and sarcopenia-related outcomes evaluated. Longitudinal evidence and studies relating diet quality to a holistic definition of sarcopenia were lacking. Although limited and predominantly cross-sectional, the evidence consistently showed that diet quality defined by diversity and nutrient adequacy was positively associated with sarcopenia components, such as muscle mass, muscle strength, and physical performance.

## 1. Introduction

Sarcopenia is a common and serious condition among the ageing population, characterised by loss of muscle mass, strength, and physical performance [1]. For the first time in 1989, Rosenberg proposed the term “sarcopenia” to describe this age-related decrease of muscle mass [2]. Three consensus-based operational definitions of sarcopenia by the European Working Group on Sarcopenia in Older People (EWGSOP) [3], the European Society for Clinical Nutrition and Metabolism Special Interest Groups (ESPEN-SIG) [4], and the International Working Group on Sarcopenia (IWGS) [5] have been put forward more recently and are commonly accepted definitions of sarcopenia. As per the EWGSOP, an individual may have a deficit in skeletal muscle mass, along with deficits in either muscle strength or muscle performance. In the presence of the three criteria, sarcopenia is diagnosed as being severe. As per the ESPEN-SIG, skeletal muscle mass and muscle strength deficit is defined as sarcopenia. As per the IWGS, deficits in skeletal muscle mass and muscle function relating to muscle mass deficit, alone or combined with increased fat mass, are delineated as sarcopenia. By segregating muscle strength and muscle performance, the EWGSOP consensus allows for a broader description and classification of the severe conditions. The Asian Working Group for Sarcopenia (AWGS) also agrees with previous proceedings that sarcopenia should be defined as a combination of reduced muscle mass, low muscle strength, and/or low physical performance [3].

Generally, in older adults, muscle mass declines at a rate of 1.5% per year after the fifth decade, and it further increases to 3% per year in their eighth decade [6]. Most importantly, the ageing-related biological changes drive sarcopenia, resulting in physical disability, poorer health status, and higher mortality in older adults [7]. Moreover, sarcopenia triggers a substantial financial burden, with estimates of around a 34% rise in hospitalisation costs [8]. 

Increasing age and female gender are non-modifiable risks for sarcopenia. However, modifiable risk factors, such as dietary intake, may influence the changes in the body composition of the ageing population [9]. Poor diets and nutritional status are common in older adults, especially in fragile individuals. Hence addressing diet and nutrition may be helpful in both preventing and treating sarcopenia [10]. Several studies have reported that certain nutrients, particularly protein, Vitamin D, and calcium, play an essential role in preventing muscle loss among older adults [11,12,13,14]. The Mediterranean diet, which includes a larger intake of fruits and vegetables, has been linked to increased physical performance and protection against muscle wasting and sarcopenia in Mediterranean countries, Hong Kong, Japan, and France [15]. Thus, sarcopenia may be linked to dietary deficits in general and/or in specific nutrients due to reduced food intake among older adults.

One way to holistically evaluate diet quality is through the use of one of the several scoring indices to assess diets based on four aspects: (i) adequacy, (ii) variety, (iii) moderation, and (iv) overall balance, with the respective scores being totalled up [16]. The first aspect is “adequacy”, which conforms to the serving sizes of food groups. The “variety” aspect studies the overall consumption of food groups and the variety of protein sources. Scoring for “moderation” evaluates the consumption of total fat, saturated fat, dietary cholesterol, sodium, and empty calories. For the last aspect, “overall balance”, the macronutrient and fatty acid ratio intake are calculated [16]. Diet quality is used to classify an individual’s dietary intake as poor, needing improvement, or good based on the calculation of the total scores. Practicing good dietary habits corresponds to a higher diet quality score [16]. 

Various dietary metrics have been developed and used to assess diet quality, such as Diet Quality Index (DQI), Diet Quality Index-International (DQI-I), Healthy Eating Index (HEI), Alternative Healthy Eating Index (AHEI), Food Pyramid Index (FPI), Food Variety Score (FVS), and others [16,17,18]. Some developing countries have tailored the diet quality scores to align with their country-specific recommended nutrient intake and dietary guidelines [19,20]. The individual’s age, gender, and health status at different stages of the lifespan are also considered when assessing diet quality [17]. This in-depth approach interprets individuals’ overall dietary intake, nutrition deficiency, and dietary lifestyle choices [16]. Diet quality can also predict nutrition-related health outcomes to set effective public health and nutrition interventions at a community or population level. Older adults are specifically at risk for poor diet quality owing to changes related to ageing and increased morbidity [21].

Despite high heterogeneity across primary studies, a meta-analysis estimated a global prevalence of sarcopenia at 10% [22]. The analysis points explicitly to a higher prevalence among non-Asian populations compared to Asian populations, with a higher prevalence among women than in men [22]. The reported prevalence of sarcopenia in developing countries ranges from 9.9% to 40.4%, depending on the diagnostic criteria used [23,24,25]. In Japan, sarcopenia was prevalent among 9.9% of the older adults as per the AWGS standard [26]. The low prevalence could be attributed to its economic and technological advancement and high prevalence of literacy. A similar study among older Chinese adults documented a sarcopenia prevalence of 14% [27]. This variation could also be determined by the characteristics of the population, the methodology used to assess parameters of sarcopenia, or a combination of both factors [24,25]. With rapid increases in the ageing population, sarcopenia is likely to increasingly be a significant public health concern driven by the rapid demographic transitions resulting from high birth and high death rates to low birth rates and low death rates [28]. This demographic transition is expectedly paralleled by the increasing prevalence of sarcopenia in many developing countries with large and rapidly ageing populations [29,30]. Despite its importance, data on sarcopenia prevalence is scarce, with a specific paucity of data in developing countries [6]. While diet is a potential modifiable risk factor for sarcopenia [10,11,12,13,14,15], the impact of diet quality on sarcopenia is poorly understood in developing countries [30]. Hence, we conducted a systematic review of studies exploring the associations between diet quality and sarcopenia measures in developing economies. 

## 2. Materials and Methods

### 2.1. Study Design

We conducted a systematic review using the updated Preferred Reporting Items for Systematic Reviews and Meta-Analyses (PRISMA) 2020 statement [31] and checklist (Supplementary Table S1). The systematic review protocol has been registered with the International Prospective Register of Systematic Reviews (PROSPERO) (CRD42021290197).

### 2.2. Search Strategy

A systematic literature search of peer-reviewed articles was conducted using the following electronic bibliographic databases: OVID Medline, PubMed, CINAHL Plus, Scopus, Embase, and ScienceDirect (Supplementary Table S2). The search strategy was built using the following keywords and Boolean operators: (‘diet quality’ OR ‘diet quality index’ OR ‘healthy eating index’ OR ‘food variety’ OR ‘diet diversity’ OR ‘diet diversity score’) AND (‘sarcopenia*’ OR ‘muscular atrophy’ OR ‘muscle fatigue’ OR ‘muscle strength’ OR ‘muscle mass’ OR “physical performance”). The search was conducted from a journal’s inception to 31 December 2021 and limited to “human studies” where possible. No language restriction was imposed. 

### 2.3. Study Selection

The study selection process used a two-staged screening approach with the Covidence platform (Veritas Health Innovation, Australia). Duplicate records were automatically removed in Covidence. The first screening step was based on the titles and abstracts, according to the eligibility criteria. The second step explored the full text of the papers for their methods and results. Two authors conducted both screening processes independently, and a third author resolved any arising conflicts. 

We included all studies (interventional or observational) that have reported a relationship between overall diet quality (such as healthy eating index, diet diversity, or food variety) and at least one of the following measures of sarcopenia: muscle strength, muscle mass, or physical performance [30] in people living in developing economies as classified by the World Economic Situation and Prospects 2021 [32], regardless of their sex, age groups, ethnicity, or health status. Grey literature and non-peer-reviewed publications, such as book chapters, online abstracts, and conference proceedings, were excluded. We excluded studies using a composite score, such as lifestyle score used to describe diet quality, and studies that reported only an individual nutrient or food group. Studies that reported subjective measurements, such as perception, were also excluded. 

Manual hand-searching of the reference lists of the included studies was also performed to seek articles not identified in the database searches. 

### 2.4. Quality Assessment

Methodological quality assessments of the included studies were performed by two independent reviewers using the NIH Quality Assessment Tool for Observational Cohort and Cross-Sectional Studies [33]. The authenticity of the study was assessed using the NIH Quality Assessment Tool that encompasses 14 requirements. If a criterion was met, the requirement was answered “Yes”, and if the criterion was not met, the requirement was answered “No” or “cannot determine/not reported/not applicable”. Ultimately, the NIH Quality Assessment Tool was used to assess the overall quality rating of the observational cohort and cross-sectional studies. As the study designs included observational studies, the NIH Quality Assessment Tool was considered an appropriate tool for quality assessment.

### 2.5. Data Extraction and Synthesis

Data from the eligible papers were extracted using Google sheets by three authors. The study origin, the age of participants, study setting, diet quality measure(s) used, measures of sarcopenia, and most important findings were extracted from the final eligible papers. The data were independently reviewed and verified by two authors. No attempt was made to contact the authors for additional information. A narrative synthesis was performed on the association between overall diet quality and individual measures of sarcopenia. No meta-analysis was performed due to the heterogeneous nature of the studies and data. 

## 3. Results

### 3.1. Study Selection and Characteristics

**Figure 1:**
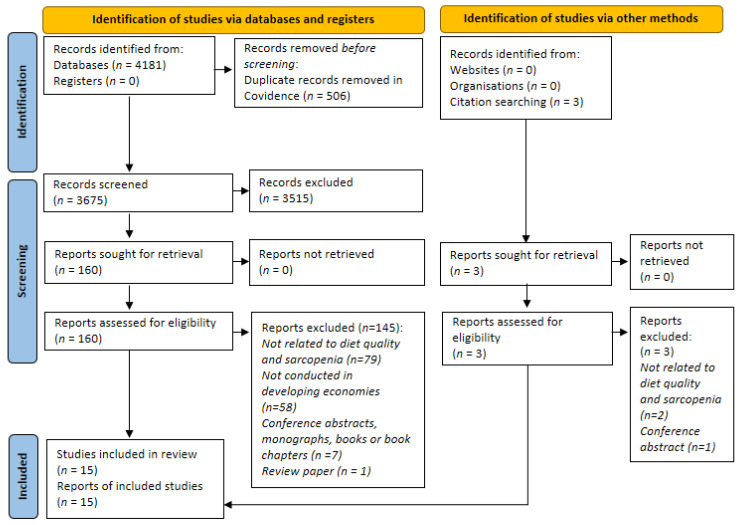
PRISMA 2020 flow chart showing the study selection process.

A total of 4181 records were retrieved from the database searches. After the removal of duplicates, 3675 titles and abstracts were screened. A total of 160 full texts were sought from this and 15 studies were finalised after screening, according to the review’s eligibility criteria. An additional three studies were found via manual searching but were excluded based on the exclusion criteria. Figure 1 presents the PRISMA flow chart of the study selection. A summary of all the studies included in the review is provided in Supplementary Table S3.

### 3.2. Study Settings and Population

Table 1 presents the summary characteristics of the included studies. Sixof the studies were conducted in South Korea [19,34,35,36,37,38], in which four articles [34,35,36,38] presented data obtained from the Korea National Health and Nutrition Examination Survey (KNHANES), a national surveillance system, while two articles [19,37] presented data obtained from the National Fitness Award. Three studies were conducted in Iran [39,40,41], followed by two studies in Brazil [42,43]. Chile [44], Hong Kong [45], Taiwan [46], and Israel [47] reported one study each. Most of the studies were published within the last 5 years (80%) [19,34,36,37,38,39,40,41,42,43,46,47], cross-sectional in design (86.7%) [19,34,35,36,37,38,39,40,41,42,43,44,47], and had a sample size larger than 2,000 respondents (40%) [34,35,36,38,39,44].While the majority of the studies (53.3%) were conducted among older adults [19,35,36,37,41,45,46,47], only two reported on respondents with specific health conditions [42,47]. Both prospective studies included older adults [46,47]. The studies predominantly included participants who were of normal weight, with six studies [38,40,41,42,43,47] having a greater proportion of overweight and obese participants.

### 3.3. Diet Quality Measures

Diet quality assessment of the population can be generally regarded to be based on (1) food and food groups, (2) nutrients, and (3) combinations of both (Table 2) [17]. Combination indices are generally more common, which was also evident in our review [34,35,39,40,41,42,45,47]. Two studies [35,45] even employed more than one combination index to assess the exposure status of their respondents. This is followed by food or food groups-based indicators (*n* = 5) [19,37,42,44,45]. Kim and colleagues (2015) [36] reported the only study that assessed nutrient adequacy, while Shin and Kim (2021) [38] reported quality assessment based on nutrient density.

The Mediterranean Diet Score (MDS), one of the most common indices used, was associated with higher consumption of plant-based foods and weakly associated with consumption of animal products among Taiwanese older adults [45]. Tepper and colleagues (2018) [47] reported higher consumption of carbohydrates, fibres, various micronutrients, and fatty acids among those with higher levels of adherence based on MDS. Similar results were reported in Brazil [43].

While most of the indices, such as Healthy Eating Index 2015 (HEI-2015), Diet Quality Index International (DQI-I), Mediterranean Diet Quality Index for children and adolescents (KIDMED), and Dietary Diversity Score (DDS), were reported in their original form [40,41,44,45,46,48], some indices were either modified or combined with other indicators. For example, an Iranian study created the Healthy Lifestyle Score (HLS), a composite score, using HEI-2015 score among other lifestyle indicators [39]. The study reported that those in the highest tertile of HLS have lower protein but higher fruit intake than those in the lowest tertile. In addition to that, the Recommended Food Score (RFS), a food-based tally that corresponds to dietary guidelines, was modified according to the needs of the local population [48] before being used to assess diet quality in the National Fitness Award Study [19,37]. 

Our synthesis also found that newer studies were more inclined to use combination indices, especially HEI-2015 [39,40,41]. In addition, we also found ESQUADA, a unique 25-item scale that has incorporated indicators of eating practice and degree of food processing [49] to assess diet quality in a Brazilian study [42]. This may indicate an evolution of the diet quality assessment to include other aspects of diet, such as eating behaviours and food choices, rather than foods alone to determine an individual’s diet quality. 

### 3.4. Study Quality

Quality assessment (Supplementary Table S4) found that all the studies’ research questions, objectives, and study population were clearly stated and defined. The participation rate of eligible persons was more than half across all the studies, resulting in low dropout and less missing data. As for the recruitment of the study subjects, the criteria for inclusion and exclusion were systematically specified among the studies. Most of the studies did not include justification of sample size [19,34,36,37,38,39,42,43,44,45,46]. There were only three studies that included sample size estimation [40,41,47]. All, except two cohorts [45,46] did not measure exposure before the outcome. Cross-sectional designs of 13 studies [19,34,35,36,37,38,39,40,41,42,43,44,47] did not allow sufficient time to see an effect, and exposures were only assessed once. As for the measured exposure, both cohort studies assessed the participants’ exposures at only one point in time [45,46]. None of the study outcome assessors was blinded to the subjects’ exposure status. As loss to follow up is a frequent issue in cohort studies, there was no loss to follow up above 20% from the beginning of the two cohort studies included in this review [45,46]. All except one study had assessed for potential covariates and adjusted them statistically. Nevertheless, Muros et al. (2016) [44] did not assess the key potential covariates.

### 3.5. Association between Diet Quality and Muscle Mass

Seven studies [19,34,36,42,43,45,46] reported on diet quality and muscle mass (Table 3). While appendicular skeletal muscle mass (ASM) was reported in three studies involving older adults [19,36,45], their findings were contradictory. Kim et al. (2015) [36] reported a significant association in females but not males, while later studies reported such association only among males [19,45]. Post-menopausal women with higher MDS were reported to have higher appendicular lean mass index (ALMI) and lumbar spine bone mineral density [43]. Silva et al. (2021) [42] demonstrated that a higher diet quality score increased mid-arm muscle circumference (MAMC). Although Lo and colleagues (2017) [46] focused on medical expenditure in more detail, they did associate higher dietary diversity with increased skeletal muscle mass index (SMMI).

### 3.6. Association between Diet Quality and Muscle Strength

A total of 10 studies showed a correlation between higher diet quality and muscle strength, with 6 studies solely investigating muscle strength. However, the findings are not uniform, with three studies demonstrating the association only in men [19,38,45] and one only in females [37]. Kim (2019) demonstrated the association between diet quality and HGS in the Korean elderly population [35]. Subsequently, Shin and Kim (2021) [38] demonstrated the associations to be significant in men below and above 65 years. Higher diet quality was associated with a 26% higher likelihood of having optimal muscle strength [40], and those in the highest tertile of diet quality score are 65% less likely to have probable-sarcopenia [41]. However, the findings may vary according to the specific nutrients studied. There were also several other uncertainties. Jeong et al. (2019) [37] presumed that HGS was the most sensitive indicator of physical performance, although other measurements did not show an association with diet quality. In addition, Muros et al. (2016) [44] investigated the association only in children, which may not be optimal in measuring sarcopenia.

### 3.7. Association between Diet Quality and Physical Performance

Physical performance was reported in four studies [37,44,45,47](Table 3). Older men with higher DQI-I scores are less likely to be sarcopenic and are seen especially in the highest quartile of DQI-I [45]. However, this association was not seen in women. In addition, a South Korean study among older adults did not find any association between physical performance indicators other than grip strength and RFS [37]. This contrasts findings by Tepper and colleagues (2018) [47]. They reported an association with adherence to a Mediterranean diet and longer distance achieved in the 6-min walk test, 10-m walk test, and Berg Balance Test in older adults with type 2 diabetes. A positive association was found between diet quality and physical fitness, which was determined using the Assessing Levels of Physical Activity health fitness test battery for children [44].

## 4. Discussion

This review systematically collates the evidence associating diet quality with sarcopenia in developing economies. The understanding of sarcopenia and its diagnostic criteria has evolved and the consensus definition was published in 2010 and 2011 [3]. All the studies included in this review were published post 2015 and showed positive associations between diet quality and accepted domains for sarcopenia assessment, including muscle mass [19,34,36,42,43,45,46], muscle strength [19,35,37,38,39,40,41,44,45,47], and physical performance [37,44,45,47]. While only one of the studies could associate diet quality with an accepted holistic definition of sarcopenia [41], all these findings agree with previous systematic reviews that evaluated the associations of diet quality with sarcopenia in older adults [30,50].

Previously, Bloom et al. [30] systematically reviewed all available evidence linking diet quality measured using a priori or posteriori dietary patterns to sarcopenia, specifically in older adults, without geographical limiters. We restricted the current review to include studies that associated a priori diet quality indices with measures of sarcopenia, including evidence from all available age groups in developing economies. By measuring adherence to a recommended dietary guideline, diet quality indices facilitate the interpretation and transferability of findings. On the other hand, the study of *posteriori* dietary patterns captures dietary intakes specific to a population, defined by its food culture and food environment. This specificity of *posteriori* dietary patterns to a particular population limits its portability. In their recent review, Jang et al. [50] included evidence only from prospective studies, including studies that measured diet quality using *a priori* or *posteriori* dietary patterns. Despite much of the evidence in our review being cross-sectional, it is interesting to note that the findings are congruent with Jang et al. [50]. 

Various measures of diet quality were used in the included studies. However, most of the studies (13/15) used indices, such as HEI and its country-specific adaptations, MDS, DQI-I, and ESQUADA, which measure nutrient adequacy and diversity. Only one study used DDS, which focused on dietary diversity in Taiwan [46], and one study measured nutrient density and adequacy with INQ [38]. Nutrient adequacy indices are commonly used to study associations with non-communicable diseases, whereas diet diversity indices are more commonly used in food-insecure settings [51]. Therefore, much of the evidence relates dietary quality as defined by nutrient adequacy to sarcopenia or its components. In the absence of nutrient sufficiency, dietary diversity may not improve muscle mass, strength, and physical performance.

Even among the nutrient adequacy indices included in this review, there are slight differences between these metrics in measuring diet quality. RFS weights fruits and vegetables highly compared to protein and calcium sources, such as milk and fish. Meat is not included in the RFS score. In comparison, MDS provides higher weightage to dairy [52]. DQI-I also uses weights to score food, assuming its nutritional importance proportionally. However, the relative importance of protein and calcium sources is comparable with vegetables and fruits having a slightly higher weight due to separate scores for fruit and vegetable intake adequacy and an additional adequacy component for vitamin C intake [53]. The applicability of such standardised weightings for food groups in diet quality indices, across scenarios, has been debated [54]. Despite these subtle differences, the studies’ results in this review consistently show benefits of improved diet quality in reducing sarcopenia related changes in muscle mass, strength, and physical function.

### 4.1. Food Groups Associated with Sarcopenia

While overall higher diet quality scores reduced the likelihood of sarcopenia, specific food groups have been indicated in a few studies. These include food sources rich in protein and calcium that have well-established roles in reducing sarcopenia and improving lean body mass and physical performance [54,55]. For instance, adherence to “snack-drink milk and vegetable patterns” was shown to reduce sarcopenia over 4 years in older men and women [45]. Interestingly, two of the studies included in the review inversely associated fruits and vegetables with measures of sarcopenia [40,45]. It was earlier shown that increased fruit and vegetable consumption modestly increases grip strength in healthy older adults [56]. 

One of the included studies also showed that higher adherence to the Mediterranean diet reduced the likelihood of low bone mineral density in post-menopausal women [43]. Apart from its emphasis on healthy proteins and fats, Mediterranean diets emphasise on adequate intakes of fruits and vegetables. Many plausible biological mechanisms have been proposed to explain the role of fruits and vegetables in bone health. These are ascribed to: (i) their fibre content that could potentially improve calcium absorption through lowered pH in the colon, (ii) their antioxidant potential relating to their vitamin and flavonoid content having a role in reducing bone resorption and muscle loss, and (iii) the complex role of vitamin B in bone mineral density and content [57]. Accordingly, evidence from various cohorts associates improved bone health with increasing intakes of fruits and vegetables [58,59,60].

Furthermore, lean muscle mass seems to facilitate bone mass accrual and retention with positive correlations between muscle mass and bone size and strength. Additionally, diminished muscle quality is associated with diminished bone quality. Sarcopenia itself increases the risk for fragility fractures and lower bone density in several studies [61]. This close interaction between muscle and bone suggests that therapies, including dietary interventions for sarcopenia and bone health, may be closely related. Thus, improving diet quality may improve muscle mass, strength, and bone health.

There is scant literature relating the inverse associations of negative nutrients, such as added sugars and saturated fats, with sarcopenia and muscle strength. Two studies included in this review indicated their involvement in sarcopenia. Lower added sugar [40,41] and saturated fat [41] intakes were associated with higher HGS. The mechanism through which saturated fat and sugar reduce HGS could be multipronged. Within the narrow intra-individual caloric intake variations, additional calories from sugar and saturated fat may replace protein intake [62,63]. Additionally, increased consumption of sugar and saturated fat and the ensuing lower diet quality are usually associated with other unhealthy lifestyle patterns, such as increased stress, lack of physical activity, and inadequate sleep [64,65]. These interactions could result in the negative associations between added sugar and saturated fat intake on muscle strength.

Additionally, excessive added sugar and saturated fat intakes have been associated with increased adiposity [66]. This becomes specifically pertinent to the developing countries that account for 67% of the world’s obese individuals [67]. Furthermore, obesity prevalence trends in these nations are expected to surpass those reported in developed countries [68]. This increase in obesity prevalence is more rapid in lower-middle and low-income countries and territories than in those with high income [69]. These findings gain importance given that sarcopenia or low muscle mass may commonly be accompanied by increased adiposity in older adults. This condition is referred to as sarcopenic obesity. It is increasingly noticed in the elderly and is associated with a higher risk of physical disability [70,71].

### 4.2. Difference in the Association between Males and Females

Several cross-sectional studies reported gender differences in the association of diet quality with sarcopenia measures. Studies have reported benefits of diet quality in women but not men for body composition and HGS. Kim et al. (2015 and 2017) [34,36] showed that the strength of a positive association between diet quality (KHEI, DASH, and aMED) and muscle mass or body composition was greater in Korean women than men. Jeong et al. [37] indicated that RFS was positively associated with muscle strength measured by HGS in Korean women but not men. Silva et al. (2019) [43], in a cross-sectional study in Brazil that included only women, also showed benefits of diet quality (MDS) on body composition.

On the contrary, Jung et al. [19] demonstrated positive associations between diet quality and muscle mass in men, but not women. Similarly, Chan et al. [45] showed that higher diet quality (DQI-I) was associated with lower odds of sarcopenia prevalence in older Chinese men but not women at baseline. However, in their subsequent 4-year longitudinal analysis, none of the dietary patterns was associated with sarcopenia in men and women. Silva et al. (2021) [42] showed that higher diet quality measured using ESQUADA improved body composition in Brazilian men and women. Shin and Kim (2021) [38] showed that higher diet quality (lower INQ scores) was associated with improved HGS in his men only cohort.

Differences in diet quality indices could not account for these anomalies, since studies that measured diet quality similarly had conflicting results. Age did not seem to be instrumental in these differences, with conflicting results reported within similar age groups. In this context, it is interesting that the association between muscle mass and muscle strength in older adults is moderated by obesity, with muscle mass being a significant predictor of muscle strength only in non-obese participants [72]. The majority of the studies in this review had participants with normal-weight, with approximately 40% of the studies having participants with overweight and obesity. The studies also differed in the proportion of male and female participants. Therefore, it is plausible that gender differences in adiposity could have an implication on the association between diet quality and sarcopenic measures.

Furthermore, these anomalies in gender-based differences in the association of diet quality with measures of sarcopenia may be an artefact of the different adjustments made to the study model. In their article, Jung et al. (2019) [19] discussed potential reasons to explain these gender differences, many of which are findings from cross-sectional studies. One of the reasons they propose is gender differences in smoking and alcohol consumption that may confound the relationship. Chan et al. (2016) [45] and Kim et al. (2015) [36] also implied that differences in lifestyle factors, such as lower physical activity in women, might explain gender differences in the association in cross-sectional studies. Jung et al. [19] and Jeong et al. [37] further suggest that gender differences may also be explained by hormone-driven differences in the absolute values of muscle mass and function and the rate of decrease with age in muscle mass and function. Kim et al. (2017) [34] attributed gender differences to their analysis not accounting for the carbohydrate quality (glycaemic index) and their confounding effects. Kim et al. (2015 and 2017) [34,36] used a different set of elaborate adjustments for men and women. In contrast, Silva et al. (2021) [42], who showed no differences between men and women in their analysis of the association between diet quality using ESQUADA with lean muscle mass in Brazil, used the same set of adjustments for men and women. 

These variations may make direct comparisons challenging. Nevertheless, the need to further understand these gender differences has been acknowledged by several authors who observed the anomalies [19,34,36,37,45]. Thus, future studies evaluating the association of diet quality with sarcopenia should consider the variables to be measured in identifying the ideal adjustment sets. Different studies have considered different confounders; it is essential to solidify this association’s conceptual framework. Caution should prevent collider bias and errors arising from over adjustment.

### 4.3. Limitations and Strengths

The authors acknowledge several limitations in assuming causality between diet quality and sarcopenia. First, this review did not restrict the inclusion criteria of the studies by study design. Only 2/15 included findings were from prospective cohort studies [45,46]. Given that majority of the studies were cross-sectional [19,34,36,37,38,39,41,42,44,46,72], establishing a direct causality between diet quality and measures of sarcopenia is theoretically limited. However, plausible biological mechanisms, including the established role of adequate protein and calcium intakes and the mechanistic role of vegetables in preventing age-associated declines in muscle and bone mass, validate these findings. Secondly, very few studies [41,45] used a holistic diagnosis of sarcopenia, including the three aspects of reduced muscle mass, low muscle strength, and/or low physical performance as recommended by various consensus-based definitions [3,4,5]. This limits the ability of the majority of the studies to identify the varying dimensions and severity of the condition. Thirdly, while the sample sizes range between 110 to over 5000 individuals, the evidence obtained is limited. It comes from a few countries and includes very few nationally representative datasets. Most of the evidence comes from Korea (*n* = 6) and Iran (*n* = 3). There is a glaring lack of evidence from South Asia, Southeast Asia, the Middle-east, Africa, South America, Latin America, and the Caribbean. Therefore, adherence to the national dietary guidelines of several developing countries and its effect on sarcopenia remains poorly understood. The heterogeneity in the studies also precludes precisely quantifying the relationship between diet quality and sarcopenia measures. Additionally, given the associations between socio-economic factors, including income, education, access to healthcare, and diet quality, the effects of residual confounding cannot be discounted. This reiterates the need to carefully design future studies to measure the effects of diet quality on sarcopenia accurately.

Nevertheless, this review scopes the current evidence for diet quality in preventing sarcopenia among older adults in developing economies. It is noted that the association between improved diet quality and decreased risk of sarcopenia or its constituent characteristics is observable across the study locations, irrespective of the tools used to measure diet quality. These findings are vital given that socio-economic status is an important determinant of diet quality in developing economies, as seen in Brazil, China, and Iran [73]. Given that sarcopenia is a significant determinant of healthy longevity and quality of life [34,74,75,76], the findings of this review show the potential importance of diet quality in improving healthy longevity in older adults. This raises the need to make quality diets affordable to have a public health impact as life expectancy increases in populations of developing countries.

## 5. Conclusions

Limited evidence is available from the developing countries to evaluate the association between diet quality and sarcopenia. Specifically, longitudinal evidence for the role of diet quality in the development of sarcopenia is lacking. Currently, available data indicates that diet quality indices focusing on nutrient adequacy have consistent positive associations with accepted domains for sarcopenia assessment, including muscle mass, muscle strength, and physical performance, irrespective of the study location. While many developing countries are poorly represented in the current literature, the existing evidence for association is unequivocal. It points to the potential benefits of improving diet quality to reduce the prevalence of sarcopenia and promote healthy ageing. 

## Figures and Tables

**Table 1:** Characteristics of studies included in the review (*n* = 15).

		*N* (%)	References
Year of publication	2015	1 (6.7)	[36]
	2016	2 (13.3)	[44,45]
	2017	2 (13.3)	[34,46]
	2018	1 (6.7)	[47]
	2019	4 (26.7)	[19,35,37,43]
	2020	2 (13.3)	[39,40]
	2021	3 (20.0)	[38,40,41]
Country	South Korea	6 (40.0)	[19,34,35,36,37,38]
	Iran	3 (20.0)	[39,40,41]
	Brazil	2 (13.3)	[42,43]
	Chile	1 (6.7)	[44]
	Hong Kong	1 (6.7)	[45]
	Taiwan	1 (6.7)	[46]
	Israel	1 (6.7)	[47]
Study design	Cross-sectional	13 (86.7)	[19,34,35,36,37,38,39,40,41,42,43,44,47]
	Prospective cohort	2 (13.3)	[45,46]
Sample size	<500	4 (26.7)	[39,41,43,45]
	501–1000	3 (20.0)	[19,37,44]
	1001–2000	2 (13.3)	[42,46]
	>2001	6 (40.0)	[34,35,36,38,40,45]
Age of study population	Children (<18 years)	1 (6.7)	[44]
	Adult men and women	4 (26.7)	[34,39,40,42]
	Adult women only	1 (6.7)	[43]
	Adult men only	1 (6.7)	[38]
	Older adults (≥65 years)	8 (53.3)	[19,35,36,37,41,45,46,47]
Body weight status- based on BMI	Predominantly underweight	0(0)	
	Predominantly normal weight	8 (53.3)	[19,35,36,37,39,44,45]
	Predominantly overweight and obese	6 (40.0)	[38,40,41,42,43,47]
	Not reported	1 (6.7)	[34]
Health condition	None	13 (85.7)	[19,34,35,36,37,38,39,40,41,42,45,46]
	Postmenopausal	1 (6.7)	[43]
	Type 2 diabetes	1 (6.7)	[47]

**Table 2:** Diet quality measures reported in the included studies (*n* = 15).

Category	Measures	*n*	References
Food/food group-based diversity indicators	Recommended Food Score (RFS)	2	[19,37]
	Mediterranean Diet Quality Index for children and adolescents (KIDMED)	1	[44]
	Dietary Diversity Score (DDS)	1	[46]
	Diet Quality Scale (ESQUADA)	1	[42]
Nutrient-based indicators	Dietary reference intakes for Koreans (KDRI)	1	[36]
	Index of Nutritional Quality (INQ)	1	[38]
Combination indices	Healthy Eating Index 2015 (HEI-2015)	3	[39,40,41]
	Mediterranean Diet Score (MDS)	3	[43,45,47]
	Diet Quality Index International (DQI-I)	2	[34,45]
	Alternate Mediterranean Diet (aMED)	1	[35]
	Dietary Approach to stop Hypertension (DASH) score	1	[35]
	Korean Health Eating Index (KHEI)	1	[35]

**Table 3:** Association between diet quality and selected sarcopenia measures (*n* = 15).

Measure	*n*	Variable	All	Males	Females	References
Muscle mass	7	Appendicular skeletal muscle mass(ASM)		● ●	●	[19,36,45]
		Multiple body composition abnormalities			●	[34]
		Skeletal muscle mass index (SMMI)	●			[46]
		Appendicular lean mass index (ALMI)			●	[43]
		Mid Arm Muscle Circumference(MAMC)	●			[42]
Muscle strength	10	Hand-grip strength (HGS)	● ● ● ● ●	● ● ●	●	[19,35,37,38,39,40,41,44,45]
		Grip and pinch strength	●			[47]
Physical performance	4	Gait speed		●		[45]
		Timed-get-up-and-go	●			[37,47]
		Assessing Levels of Physical Activity health fitness test battery	●			[44]
		6-m walk, 10-m walk, Berg Balance Scale (BBS), Four Square Step Test (FSST), 30-s chair stand	●			[47]

Note: ● = positive association between diet quality and specific measures of sarcopenia.

## Data Availability

Not applicable.

## Supplementary Materials

The following supporting information can be downloaded at: https://www.mdpi.com/article/10.3390/nu14040868/s1, Table S1: PRISMA checklist; Table S2: Search strategy in Ovid Medline; Table S3: Summary of all included studies (*n* = 14); Table S4: Assessment of study quality.
