# Supplementary material for: Diet Quality and Measures of Sarcopenia in Developing Economies: A Systematic Review
Source: Nutrients. 2022 Feb 18;14(4):868. doi: 10.3390/nu14040868 (PMC8874949; doi:10.3390/nu14040868)
Supplement: Supplementary file 1 [file nutrients-14-00868-s001.zip › nutrients-1556144-supplementary.pdf]

Supplementary Tables

Table S1. PRISMA 2020 checklist

Section and Topic	Item #	Checklist item	Location where item is reported
**TITLE**			
Title	1	Identify the report as a systematic review.	Page 1
**ABSTRACT**			
Abstract	2	See the PRISMA 2020 for Abstracts checklist.	Page 1
**INTRODUCTION**			
Rationale	3	Describe the rationale for the review in the context of existing knowledge.	Page 1-3
Objectives	4	Provide an explicit statement of the objective(s) or question(s) the review addresses.	Page 3
**METHODS**			
Eligibility criteria	5	Specify the inclusion and exclusion criteria for the review and how studies were grouped for the syntheses.	Page 3
Information sources	6	Specify all databases, registers, websites, organisations, reference lists and other sources searched or consulted to identify studies. Specify the date when each source was last searched or consulted.	Page 3
Search strategy	7	Present the full search strategies for all databases, registers and websites, including any filters and limits used.	Page 3
Selection process	8	Specify the methods used to decide whether a study met the inclusion criteria of the review, including how many reviewers screened each record and each report retrieved, whether they worked independently, and if applicable, details of automation tools used in the process.	Page 3
Data collection process	9	Specify the methods used to collect data from reports, including how many reviewers collected data from each report, whether they worked independently, any processes for obtaining or confirming data from study investigators, and if applicable, details of automation tools used in the process.	Page 4
Data items	10a	List and define all outcomes for which data were sought. Specify whether all results that were compatible with each outcome domain in each study were sought (e.g. for all measures, time points, analyses), and if not, the methods used to decide which results to collect.	Page 4
	10b	List and define all other variables for which data were sought (e.g. participant and intervention characteristics, funding sources). Describe any assumptions made about any missing or unclear information.	Page 4
Study risk of bias assessment	11	Specify the methods used to assess risk of bias in the included studies, including details of the tool(s) used, how many reviewers assessed each study and whether they worked independently, and if applicable, details of automation tools used in the process.	Page 4
Effect measures	12	Specify for each outcome the effect measure(s) (e.g. risk ratio, mean difference) used in the synthesis or presentation of results.	N/A
Synthesis methods	13a	Describe the processes used to decide which studies were eligible for each synthesis (e.g. tabulating the study intervention characteristics and comparing against the planned groups for each synthesis (item #5)).	Page 4
	13b	Describe any methods required to prepare the data for presentation or synthesis, such as handling of missing summary statistics, or data conversions.	N/A
	13c	Describe any methods used to tabulate or visually display results of individual studies and syntheses.	N/A
	13d	Describe any methods used to synthesize results and provide a rationale for the choice(s). If meta-analysis was performed, describe the model(s), method(s) to identify the presence and extent of statistical heterogeneity, and software package(s) used.	N/A

Table S1. PRISMA 2020 checklist

Section and Topic	Item #	Checklist item	Location where item is reported
	13e	Describe any methods used to explore possible causes of heterogeneity among study results (e.g. subgroup analysis, meta-regression).	N/A
	13f	Describe any sensitivity analyses conducted to assess robustness of the synthesized results.	N/A
Reporting bias assessment	14	Describe any methods used to assess risk of bias due to missing results in a synthesis (arising from reporting biases).	N/A
Certainty assessment	15	Describe any methods used to assess certainty (or confidence) in the body of evidence for an outcome.	N/A
**RESULTS**			
Study selection	16a	Describe the results of the search and selection process, from the number of records identified in the search to the number of studies included in the review, ideally using a flow diagram.	Page 4
	16b	Cite studies that might appear to meet the inclusion criteria, but which were excluded, and explain why they were excluded.	Page 4
Study characteristics	17	Cite each included study and present its characteristics.	Table S3, Page 5-6
Risk of bias in studies	18	Present assessments of risk of bias for each included study.	Table S4, Page 6-7
Results of individual studies	19	For all outcomes, present, for each study: (a) summary statistics for each group (where appropriate) and (b) an effect estimate and its precision (e.g. confidence/credible interval), ideally using structured tables or plots.	N/A
Results of syntheses	20a	For each synthesis, briefly summarise the characteristics and risk of bias among contributing studies.	Page 7-8
	20b	Present results of all statistical syntheses conducted. If meta-analysis was done, present for each the summary estimate and its precision (e.g. confidence/credible interval) and measures of statistical heterogeneity. If comparing groups, describe the direction of the effect.	N/A
	20c	Present results of all investigations of possible causes of heterogeneity among study results.	N/A
	20d	Present results of all sensitivity analyses conducted to assess the robustness of the synthesized results.	N/A
Reporting biases	21	Present assessments of risk of bias due to missing results (arising from reporting biases) for each synthesis assessed.	N/A
Certainty of evidence	22	Present assessments of certainty (or confidence) in the body of evidence for each outcome assessed.	N/A
**DISCUSSION**			
Discussion	23a	Provide a general interpretation of the results in the context of other evidence.	Page 8-10
	23b	Discuss any limitations of the evidence included in the review.	Page 10
	23c	Discuss any limitations of the review processes used.	Page 10
	23d	Discuss implications of the results for practice, policy, and future research.	Page 8-10
**OTHER INFORMATION**			

PRISMA 2020 Checklist

Table S1. PRISMA 2020 checklist

Section and Topic	Item #	Checklist item	Location where item is reported
Registration and protocol	24a	Provide registration information for the review, including register name and registration number, or state that the review was not registered.	Page 3
	24b	Indicate where the review protocol can be accessed, or state that a protocol was not prepared.	Page 3
	24c	Describe and explain any amendments to information provided at registration or in the protocol.	N/A
Support	25	Describe sources of financial or non-financial support for the review, and the role of the funders or sponsors in the review.	Page 11
Competing interests	26	Declare any competing interests of review authors.	Page 11
Availability of data, code and other materials	27	Report which of the following are publicly available and where they can be found: template data collection forms; data extracted from included studies; data used for all analyses; analytic code; any other materials used in the review.	Table S3

From: Page MJ, McKenzie JE, Bossuyt PM, Boutron I, Hoffmann TC, Mulrow CD, et al. The PRISMA 2020 statement: an updated guideline for reporting systematic reviews. BMJ 2021;372:n71. doi: 10.1136/bmj.n71

For more information, visit: http://www.prisma-statement.org/

Table S2. Database search strategies*

EMBASE		
**#**	**Keyword**	**Results**
1	diet quality.mp.	6899
2	diet quality index.mp.	641
3	healthy eating index.mp.	2635
4	food variety.mp.	333
5	diet diversity.mp.	399
6	diet diversity score.mp.	65
7	1 or 2 or 3 or 4 or 5 or 6	8457
8	sarcopenia/ or sarcopeni*.mp.	21588
9	muscular atrophy.mp. or muscular atrophy/	37664
10	muscle fatigue.mp. or muscle fatigue/	15611
11	muscle strength.mp. or muscle strength/	82146
12	muscle.mass.mp. or muscle mass/	43939
13	physical performce.mp. or Physical Functional Performance/	30396
14	8 or 9 or 9 or 10 or 11 or 12 or 13	194677
15	7 and 14	106
16	limit 15 to humans	102

CINAHL+		
**#**	**Keyword**	**Results**
S1	diet quality OR diet quality index OR healthy eating index OR food variety OR diet diversity OR diet diversity score	6165
S2	(sarcopenia or sarcopenic) OR muscular atrophy OR muscle fatigue OR muscle strength OR muscle mass OR physical performance	54224
S3	S1 AND S2	86

PUBMED		
**#**	**Keyword**	**Results**
1	(diet quality) OR (diet quality index) OR (healthy eating index) OR (food variety) OR (diet diversity) OR (diet diversity score)	131322
2	(sarcopenia) OR (sarcopenic) OR (muscular atrophy) OR (muscle fatigue) OR (muscle strength) OR (muscle mass) OR (physical performance)	289799
3	((diet quality) OR (diet quality index) OR (healthy eating index) OR (food variety) OR (diet diversity) OR (diet diversity score)) AND ((sarcopenia) OR (sarcopenic) OR (muscular atrophy) OR (muscle fatigue) OR (muscle strength) OR (muscle mass) OR (physical performance))	3096
4	((diet quality) OR (diet quality index) OR (healthy eating index) OR (food variety) OR (diet diversity) OR (diet diversity score)) AND ((sarcopenia) OR (sarcopenic) OR (muscular atrophy) OR (muscle fatigue) OR (muscle strength) OR (muscle mass) OR (physical performance)). Filters: Humans	2265

SCIENCEDIRECT		
**#**	**Keyword**	**Results**
	("diet quality" OR "diet quality index" OR "healthy eating index" OR "food variety" OR "diet diversity" OR "diet diversity score") AND (sarcopenia OR	1595

	"muscular atrophy" OR "muscle fatigue" OR "muscle strength" OR "muscle mass" OR "physical performance")	

SCOPUS		
#	Keyword	Results
1	(TITLE-ABS-KEY ("diet quality") OR (TITLE-ABS-KEY ("diet quality index") OR (TITLE-ABS-KEY ("healthy eating index") OR (TITLE-ABS-KEY ("food variety") OR (TITLE-ABS-KEY ("diet diversity") OR (TITLE-ABS-KEY ("diet diversity score")	171349
2	(TITLE-ABS-KEY ("sarcopeni*") OR (TITLE-ABS-KEY ("muscular atrophy") OR (TITLE-ABS-KEY ("muscle fatigue") OR (TITLE-ABS-KEY ("muscle strength") OR (TITLE-ABS-KEY ("muscle mass") OR (TITLE-ABS-KEY ("physical performance")	8635
3	((TITLE-ABS-KEY ("diet quality") OR (TITLE-ABS-KEY ("diet quality index") OR (TITLE-ABS-KEY ("healthy eating index") OR (TITLE-ABS-KEY ("food variety") OR (TITLE-ABS-KEY ("diet diversity") OR (TITLE-ABS-KEY ("diet diversity score")) AND ((TITLE-ABS-KEY ("sarcopeni*") OR (TITLE-ABS-KEY ("muscular atrophy") OR (TITLE-ABS-KEY ("muscle fatigue") OR (TITLE-ABS-KEY ("muscle strength") OR (TITLE-ABS-KEY ("muscle mass"))	86
4	((TITLE-ABS-KEY ("diet quality") OR (TITLE-ABS-KEY ("diet quality index") OR (TITLE-ABS-KEY ("healthy eating index") OR (TITLE-ABS-KEY ("food variety") OR (TITLE-ABS-KEY ("diet diversity") OR (TITLE-ABS-KEY ("diet diversity score")) AND ((TITLE-ABS-KEY ("sarcopeni*") OR (TITLE-ABS-KEY ("muscular atrophy") OR (TITLE-ABS-KEY ("muscle fatigue") OR (TITLE-ABS-KEY ("muscle strength") OR (TITLE-ABS-KEY ("muscle mass")) AND (LIMIT-TO (EXACTKEYWORD, "Human"))	74

Ovid Medline		
#	Keyword	Results
1	diet quality.mp.	4533
2	diet quality index.mp.	404
3	healthy eating index.mp.	1674
4	food variety.mp.	231
5	diet diversity.mp.	245
6	diet diversity score.mp.	37
7	1 or 2 or 3 or 4 or 5 or 6	5512
8	sarcopenia/ or sarcopeni*.mp.	10659
9	muscular atrophy.mp. or muscular atrophy/	19495
10	muscle fatigue.mp. or muscle fatigue/	10843
11	muscle strength.mp. or muscle strength/	37056
12	muscle.mass.mp. or muscle mass/	18410
13	physical performance.mp. or physical functional performance/	11042
14	8 or 9 or 9 or 10 or 11 or 12 or 13	91702
15	7 and 14	61
16	limit 15 to humans	59

Note: *As of 31 December 2021

Table S3. Summary of all included studies

	Study, Country	Study design	Health condition	Sample size, F/M	Age (years)	Diet quality measures	Adiposity	Other measures of obesity	Sarcopenia measures	Main findings
1	Kim et al (2015) [36]<br><br>Republic of Korea	Cross-sectional	None	3285<br><br>1486 M; 1799 W	≥65	Dietary reference intakes for Koreans (KDRIs)	BMI was categorised as: (i) normal (<25.0 kg/m²) and (ii) overweight (≥25.0 kg/m²)	Body composition (DEXA)	ASM	• Women consuming recommended levels of vegetables (OR = 0.52, 95% CI = 0.30–0.89), engaging in aerobic exercise (OR = 0.62, 95% CI = 0.39–1.00) and having >3 healthy behaviours (OR=0.45, 95% CI = 0.23-0.87) had a significantly lower likelihood of low muscle mass than those who did not meet the recommended levels. Such significant associations were not found in men.<br>• 75.8% men and 60% of women had a BMI below 25 kg/m². 24.2% of males and 39.9% of females had a BMI above 25 kg/m².
2	Chan et al (2016) [45]<br><br>Hong Kong	Prospective cohort	None	3957<br><br>1979 W; 1978 M	Mean: 76.2 (sarcopenic); 72.2 (non-sarcopenic)	DQI-I, MDS	BMI was kept as a continuous variable.	No additional measures	ASM, HGS, Gait speed	• Lower odds of sarcopenia in older men with higher DQI-I (AOR=0.50, 95% CI=0.31-0.81), "vegetables-fruits" dietary pattern (AOR=0.60, 95% C=0.36-0.99) and "snacks-drinks-milk products" dietary pattern (AOR=0.41, 95% CI=0.24-0.70) scores at baseline.<br>• Women with sarcopenia were more likely to have

										younger age, lower BMI, lower PASE, more chronic diseases, and live alone than women without sarcopenia. There was no association between all dietary pattern scores and the likelihood of being sarcopenic in women at baseline.<br>● None of the dietary patterns was associated with the presence of sarcopenia after 4 years in both men and women after a longitudinal analysis was done.<br>● The mean BMI (kg/m^2) was 23.9 for non sarcopenic adults and 20.6 for sarcopenic adults.<br>● Mean BMI (kg/m^2) for sarcopenic men and women were 20.7 and 20.5.<br>● The average BMI (kg/m^2)for non sarcopenic men and women were 23.7 and 24.1.
3	Muros et al (2016) [44]<br><br>Chile	Cross-sectional	None	515<br><br>260 M; 255 F	Mean: 10.6$\pm$0.5	KIDMED	BMI categorised as: healthy, overweight , obese	Weight, WC, skinfolds (triceps and subscapular), body fat percentage (Slaughter equation)	HGS	● Adherence to the MD was negatively associated with body fat (r =-0.302) and subscapular skinfold thickness (r =-0.329), and positively associated with physical activity scores (PAQ-C) (r=0.277), self-esteem (r=0.301), self-concept (r=0.234) and physical fitness especially explosive power of the legs

										(r=0.355). Adherence to MD was positively correlated in HGS in boys (r=0.323), and negatively correlated with screen time in girls (r=-0.511).<br>● Higher adherence to the diet was observed among children in the highest tertile of physical activity, in healthy or overweight children compared with obese children, and in children with moderate–high levels of self-esteem.<br>● BMI (mean ± SD) was 22.5 ± 3.6kg/m².
4	Kim et al (2017) [34]<br><br>Republic of Korea	Cross-sectional	None	6129<br><br>2579 M; 3550 F	Mean: 60.8±8.5 (M); 61.9±9.1 (F)	DQI-I	No BMI measure used	Body composition abnormalities were defined as:<br>0 (normal; without low bone mass, low muscle mass, or obesity)<br>1 (having one of the components)<br>2 (having two of the components), and<br>3 (OSO; having all	Multiple body composition abnormalities: number of osteopenia/ osteoporosis, muscle mass loss (ASM), or obesity.	● In women, the DQI-I score was inversely associated with a higher number of body composition abnormalities.<br>● Lower likelihood for osteosarcopenic obesity in highest tertile of DQI-score compared to the lowest (OR=0.54, 95% CI=0.32-0.92). Such significant association was not seen in men.

								three components).		
5	Lo et al (2017) [46]<br><br>Taiwan	Prospective cohort	None	1337<br><br>689 M; 648 F	≥65	DDS	BMI categorised as: underweight, normal weight, overweight, obese	No additional measure	Skeletal muscle mass index (SMMI)	• The participants with high-risk group (low SMMI) and low DDS had high mortality rate and fewer outpatient (14%), preventive care (19%), and dental (40%) service visits, but more emergency department visits (18%) and hospital stays (102%) compared with those in the low-risk group who had a high DDS.<br>• Similar patterns were observed for the corresponding medical expenditure.<br>• BMI (mean ± SD) was 23.7 ± 3.65 kg/m^2.
6	Tepper et al (2018) [47]<br><br>Israel	Cross-sectional	Type 2 diabetes	117<br><br>71 M; 46 F	Mean: 70.6±6.5	MDS	BMI was kept as a continuous variable.	No additional measure	Timed-Get-Up-and-Go, 6-m walk, 10-m walk, Berg Balance Scale (BBS), Four Square Step Test (FSST), 30-s chair stand, grip and pinch strength,	• After controlling for covariates, higher adherence to MD (3rd tertile) was associated with longer distance achieved in the 6-min walk test in the participants aged >75 years.<br>• Similar results were obtained for the 10-m walk test and Berg test: age by MD interaction was significant, indicating that higher speed of walking was associated with MD

									Functional Independence Measure and the Frenchay activity index	only in the older age (>75 years).<br>● Higher Berg score was associated with higher adherence to MD only in the older age.<br>● Significant difference in standardized grip strength score in the higher vs. lower adherence groups (low vs. high adherence −0.93 ± 0.82 vs.−0.29 ± 0.84; p = 0.03)<br>● BMI (mean ± SD) was 29.24 ± 4.79 kg/m^2.
7	Jeong et al (2019) [37]<br><br>Republic of Korea	Cross-sectional	None	622<br><br>294 M; 328 F	Mean: 71.7±4.6 (M); 71.9±5.2 (F)	RFS	BMI was kept as a continuous variable.	Body fat percentage (BIA)	2-min step test (2MST), Timed-Get-Up-and-Go test (TUG), figure-of-8 walktest (F8W), HGS, and arm curl test	● In multivariate analysis, a positive association between the RFS and grip strength (kg) (ß = 0.063, 95% CI = 0.007 to 0.119) and grip strength (%) (ß =0.105, 95% CI = 0.013 to 0.198) were in elderly women. The association remains after adjustment for physical activity. Such associations were not found in elderly men.<br>● BMI (mean ± SD) was 23.9 ± 2.7 kg/m2.<br>● BMI was 23.6 ± 2.5 kg/m^2 for male and 24.2 ± 2.8 kg/m^2 for females.
8	Jung et al (2019) [19]	Cross-sectional	None	521<br><br>263 M; 258 F	Mean: 71.9±4.9 (M); 71.4±5.3 (F)	RFS	BMI was kept as a continuous variable.	Body fat percentage (BIA)	ASM, HGS	● After adjustment for covariates, in elderly men, the low muscle mass-function (low ASM and low

	Republic of Korea									HGS) elderly men had significantly lower RFS values than their normal counterparts (p = 0.019). Such association was not found in elderly women.<br>• BMI (mean ± SD) was 23.8 ± 2.9 (kg/m2).<br>• BMI (mean ± SD) was 23.4 ± 2.8 (kg/m^2) for men and 24.2 ± 2.9 (kg/m^2) for women.
9	Kim & Kwon (2019) [35]<br><br>Republic of Korea	Cross-sectional	None	3675<br><br>1709 M; 1966 F	≥65	KHEI; aMED; DASH	BMI was kept as a continuous variable.	No additional measure	HGS	• Men and women in the 3rd tertile (associated with better diet quality) have 32-52% lower odds of low HGS compared to those in the 1st tertile (associated with poorer diet quality).<br>• BMI (kg/m^2) was reported based on tertiles in the different diet quality scores. Men with higher diet quality scores had a high BMI. There was no difference in the BMI across women.
10	Silva et al (2019) [43]<br><br>Brazil	Cross-sectional	Post-menopausal	105 F	Mean: 55.2±4.9	MDS	BMI was kept as a continuous variable.	Percentage body fat, and ALM (kg) (DEXA)	Appendicular lean mass index (ALMI)	• Women with higher adherence to the Mediterranean diet (higher MDS) had higher ALMI (Mean difference (MD)=0.296, 95% CI=0.020-0.591) and lumbar spine bone mineral density (MD=0.088, 95% CI=0.028-0.147).

										● Women with higher MDS had a lower mean BMI of 26.3 (24.2–28.1)kg/m^2 as compared to women with lower MDS with a BMI of 27.1 (23.9–31.3)kg/m^2.
11	Mohammadpour et al (2020) [39]<br><br>Iran	Cross-sectional	None	271<br><br>115 M; 155 F	Mean: T1 HLS group=35.0 ± 13.31; T2 HLS= 36.3 ± 12.3; T3 HLS=38.5 ± 14.3	HEI-2015	"Normal" BMI of 18.5-25 kg/m^2 otherwise more than 25 kg/m^2 is taken as a risk factors and receive a score of zero	Body composition analyser (BIA), WC, WHR	HGS	● In the adjusted model, higher adherence to HEI was significantly associated with higher mean muscle strength, muscle strength of the left hand and muscle strength of right hand (P_{adjust} <0.001 for all).<br>● BMI (mean± SD) of participants in tertile 3 of the HLS showed a mean BMI of 24.9 ± 4.81kg/m^2.
12	Pasdar et al (2020) [40]<br><br>Iran	Cross-sectional	None	4010<br><br>1786 M; 2224 F	Mean: 47.77±8.36	HEI-2015	BMI was kept as a continuous variable.	No additional measure	HGS	● The mean total HEI score was significantly higher in participants with an optimal HGS than in participants with a weak HGS (52.46±6.89 vs 49.54±6.97, P=0.006). Specifically, an optimal HGS was associated with higher intake of whole fruits (OR=1.01, 95% CI=1.02–1.18), and lesser consumption of added sugar (OR=1.06; 95%CI=1.01–1.12).<br>● The mean of HGS was significantly increased with the increase in the HEI-2015 score (P<0.001).

										• After adjustment for covariates, higher HEI was still associated with optimal muscle strength (OR=1.26; 95% CI= 1.02–1.62).<br>• BMI (mean+ SD) of the participants was 27±4.68 kg/m².
13	Esmaeily et al (2021) [41]<br><br>Iran	Cross-sectional	None	201<br><br>46 M; 155 F	Mean: 67.54+5.94 (probable-sarcopenic) ; 63.9+3.66 (non-sarcopenic)	HEI-2015	BMI was categorised as underweight, or overweight/obese	WC, WHR, WHtR	HGS	• Mean HEI score was higher in the normal group (60·55±9·85) compared to probable-sarcopenic (56·88±11·48) (P=0·02). However, the probable-sarcopenic subjects had lower scores of added sugars and saturated fatty acids.<br>• Participants with higher intake of total protein foods, poly- and monounsaturated fatty acids (PUFAs and MUFAs), and low intake of added sugars and saturated fats had significantly higher HGS.<br>• There was a reduction in proportions of probable-sarcopenic participants from 72.5 % in the lowest HEI quartile to 54 % in the highest quartile. Those in the top HEI quartile were 69 % less likely to have probable-sarcopenia in the adjusted model in

										comparison to those in the lowest quartile.<br>● BMI (mean+SD) of the participants was 29 kg/m².
14	Silva et al (2021) [42]<br><br>Brazil	Cross-sectional	None	1147<br><br>424 M; 713 F	Mean: 37.5 (M); 39.0 (F)	ESQUADA	BMI was categorised as thinness, eutrophy, overweight and obese.<br>BMI was also considered as continuous variable	Tricipital and subscapular skinfold, WC, MAMC	MAMC; Tricipital and subscapular skinfolds	● Higher diet quality score was associated with a reduction in tricipital skinfold (β = -0.07, 95%CI=0.13 – -0.01) and an increase in MAMC (β = 0.09, 95%CI= 0.00 – 0.18) in men and the reduction in weight (β = -0.04; 95 CI= -0.07 – -0.01), subscapular skinfold (β = -0.07, 95%CI= -0.13 – -0,00) and waist circumference in women (β = -0.06, 95%CI= -0.09 – -0.02).<br>● BMI (mean+SD) for men was 26.80 ± 5.17 kg/m² while the average BMI for women was 26.88 ± 5.33 kg/m².
15	Shin & Kim (2021)<br><br>Republic of Korea [38]	Cross-sectional	None	5748 M	Mean: 41.7	INQ	BMI was ctageorised as underweight and obese.	No additional measure	HGS	● The intake of carbohydrates, fiber, calcium, and vitamins B2 and C were significantly positively associated with a higher quartile for HGS for those aged ≥65 years (all p < .01).<br>● A significant inverse association was found between the quartiles of HGS and INQ scores among men aged ≥65 years after adjusting for all covariates

	($\beta = -0.26$, $p < .01$). This association was not found among those aged <65 years.<br>• Majority of the participants were classified as obese.<br>• There were significant associations between obesity and quartiles of HGS in men below and above 65 years (all $p<0.01$).

Note: M=males; F=females; Dietary Diversity Score = DDS; Dietary Quality Index-International=DQI-I; Mediterranean Diet Score=MDS; HEI = Healthy Eating Index; Index of Nutritional Quality = INQ; Mediterranean Diet Quality Index for children and adolescents=KIDMED; Korean Healthy Eating Index=KHEI; Alternate Mediterranean Diet=aME; Recommended Food Score = RFS; Dietary Approach to stop Hypertension = DASH; Diet Quality Scale = ESQUADA; BMI = body mass index; WC = waist circumference; WHR = waist-hip ratio; WHtR = waist-height ratio; DEXA = dual-energy X-ray absorptiometry; BIA = bioelectrical impedance analysis Mid Arm Muscle Circumference=MAMC; Appendicular skeletal muscle mass=ASM; Handgrip strength = HGS

Table S4. Assessment of study quality

Study	Chan et al (2016)	Esmaeily et al (2021)	Jeong et al (2019)	Jung et al (2019)	Kim et al (2015)	Kim et al (2017)	Kim & Kwon (2019)	Lo et al (2017)	Mohammadpour et al (2020)	Muros et al (2016)	Pasdar et al (2020)	Shin & Kim (2021)	Silva et al (2019)	Silva et al (2021)	Tepper et al (2018)
1. Clear research question / objective	Y	Y	Y	Y	Y	Y	Y	Y	Y	Y	Y	Y	Y	Y	Y
2. Specified and defined study population	Y	Y	Y	Y	Y	Y	Y	Y	Y	Y	Y	Y	Y	Y	Y
3. Participation rate	Y	Y	Y	Y	Y	Y	Y	Y	Y	Y	Y	Y	Y	Y	Y
4. Subject recruited from similar population; uniform inclusion and exclusion criteria	Y	Y	Y	Y	Y	Y	Y	Y	Y	Y	Y	Y	Y	Y	Y
5. Sample size	N	Y	N	N	N	N	N	N	N	N	Y	N	N	N	Y
6. Exposure measured before outcomes	Y	N	N	N	N	N	N	Y	N	N	N	N	N	N	N
7. Sufficient time frame	Y	N	N	N	N	N	N	Y	N	N	N	N	N	N	N
8. Different level of exposure assessment	Y	Y	Y	Y	Y	Y	Y	Y	Y	Y	Y	Y	Y	Y	Y
9. Defined, valid, reliable and consistent exposure measures	Y	Y	Y	Y	Y	Y	Y	Y	Y	Y	Y	Y	Y	Y	Y
10. Multiple exposure assessment	N	N	N	N	N	N	N	N	N	N	N	N	N	N	N
11. Defined, valid, reliable and consistent outcome measures	Y	Y	Y	Y	Y	Y	Y	Y	Y	Y	Y	Y	Y	Y	Y
12. Blinded outcome assessors	N	N	N	N	N	N	N	N	N	N	N	N	N	N	N
13. Loss to follow-up	N	NA	NA	NA	NA	NA	NA	N	NA	NA	NA	NA	NA	NA	NA
14. Confounding variables measured and adjusted statistically	Y	Y	Y	Y	Y	Y	Y	Y	Y	N	Y	Y	Y	Y	Y

N = No; Y = Yes; NA = not applicable
